# Environmental Pollution and the Risk of Developing Metabolic Disorders: Obesity and Diabetes

**DOI:** 10.3390/ijms24108870

**Published:** 2023-05-17

**Authors:** William Junior Khalil, Meriem Akeblersane, Ana Saad Khan, Abu Saleh Md Moin, Alexandra E. Butler

**Affiliations:** 1 School of Medicine, Royal College of Surgeons in Ireland Bahrain, Busaiteen 15503, Bahrain; 2 Research Department, Royal College of Surgeons in Ireland Bahrain, Busaiteen 15503, Bahrain

**Keywords:** persistent organic pollutants, atmospheric pollution, obesity, type 1 diabetes, type 2 diabetes, gestational diabetes, metabolic disorders, heavy metals, adipogenesis, diabetogenesis, obesogenic

## Abstract

To meet the increased need for food and energy because of the economic shift brought about by the Industrial Revolution in the 19th century, there has been an increase in persistent organic pollutants (POPs), atmospheric emissions and metals in the environment. Several studies have reported a relationship between these pollutants and obesity, and diabetes (type 1, type 2 and gestational). All of the major pollutants are considered to be endocrine disruptors because of their interactions with various transcription factors, receptors and tissues that result in alterations of metabolic function. POPs impact adipogenesis, thereby increasing the prevalence of obesity in exposed individuals. Metals impact glucose regulation by disrupting pancreatic β-cells, causing hyperglycemia and impaired insulin signaling. Additionally, a positive association has been observed between the concentration of endocrine disrupting chemicals (EDCs) in the 12 weeks prior to conception and fasting glucose levels. Here, we evaluate what is currently known regarding the link between environmental pollutants and metabolic disorders. In addition, we indicate where further research is required to improve our understanding of the specific effects of pollutants on these metabolic disorders which would enable implementation of changes to enable their prevention.

## 1. Introduction

Metabolic disorders are a spectrum of diseases that affect normal metabolic functioning and regulation. More than 500 metabolic disorders exist, two of the most common being diabetes mellitus and obesity [1,2]. Their growing incidence has created a rising public health concern globally.

The prevalence of obesity has seen a three-fold increase from 1975 to 2013, now afflicting 13% of the adult population worldwide [3]. Obesity in the United States (USA) increased from 30.5% to 41.9% between 1999 and 2017 in the adult population. For children and adolescents in the USA, around one in five are affected by childhood obesity [4]. Amongst individuals living in the Middle East and North Africa (MENA) region, 21% are obese and more than 33% are considered overweight [5]. In Europe, 36% of adults were considered pre-obese and 17% were considered obese in 2019 [6]. In 2021, 537 million adults worldwide were living with diabetes [7]. In the USA, 11.3% of the population was diagnosed with diabetes in 2019. Of the 37.3 million diabetic Americans identified, 90–95% had type 2 diabetes mellitus (T2D) and 1.9 million had type 1 diabetes (T1D) [8]. In 2021, in Europe, 61 million adults were diabetic. This number is even higher (1 in 6 adults, ~73 million) in the MENA region [7]. Common risk factors for both obesity and diabetes have led to many individuals developing both metabolic disorders.

Obesity is a multifactorial disorder involving the interaction of genetics, lifestyle and environment. It results in excessive adipose tissue deposition and is defined by a body mass index (BMI) greater than 30 kg/m^2^. In addition, obesity is the leading cause of metabolic syndrome and insulin resistance [9].

Type 2 diabetes is characterized by an elevated blood glucose level, a chronic hyperglycemic state caused by a combination of pancreatic β-cell loss through apoptosis and insulin resistance in peripheral tissues, such as skeletal muscle [10,11]. By contrast, type 1 diabetes is caused by autoimmune attack upon pancreatic β-cells, causing an almost complete loss of insulin production and secretion [10]. Whilst a genetic predisposition can underlie the onset of type 1 diabetes, with particular loci of interest having been identified, environmental factors may also contribute. Well-established risk factors for type 2 diabetes and obesity are a sedentary lifestyle, poor nutrition, insulin resistance, environmental factors and genetics [10].

The economic shift consequent upon the Industrial Revolution in the 19th century resulted in a global increase in population density that has necessitated a worldwide rise in production, consumption and mobility to overcome the augmented food and energy requirements [12]. To address this need, food options have become increasingly processed to increase supply and shelf life; however, this has had a detrimental effect on food quality [13]. Greater caloric intake coupled with a sedentary lifestyle disrupts energy balance in the body [14]. Whilst not based in genetics, these factors impact how our body functions at the cellular level and can lead to alterations in gene expression; this is termed the exposome, the interaction between the environment a person exists in and the individual’s genetics that combine to promote disease development [15].

As more information is gathered, new areas of concern are raised, and one that has garnered extensive interest over the years has been the impact of pollution on the human body. The atmospheric changes in air pollutants we inhale daily are a significant concern for adults but even more so for vulnerable groups, such as children and pregnant women [16]. As a result, environmental pollutants have become an area of intense research with the aim of understanding how they affect bodily systems such as the respiratory, cardiovascular and, more recently, the metabolic system.

This review aims to highlight and evaluate the extent to which pollution, in its various forms, leads to metabolic disorders such as obesity and diabetes, to mechanistically explain the vast increase in incidence of these disorders and identify potential methods of prevention.

## 2. Search Strategy

The following databases and search engines were used to find journal articles: PubMed, ScienceDirect, ReadCube Papers and Google Scholar. The databases and search engines were searched between May 2022 and August 2022 using Boolean logic in order to find articles related to environmental pollution and metabolic disorders (i.e., “Pollution and Diabetes”, “Pollution and Obesity”, “Persistent Organic Pollutants and Obesity”, “Endocrine Disruptors and Diabetes”, etc.). The references of identified articles were also examined for additional sources.

Inclusion criteria were: peer-reviewed studies that presented novel findings that dealt specifically with environmental pollution, endocrine disruptors and onset of obesity and diabetes. Papers were excluded if they: (1) focused on pollutants and other physiological disorders than metabolic syndrome (i.e., obesity and diabetes), (2) investigated the mechanisms underlying pollution-driven metabolic problems in humans without reference to specific pollutants or endocrine disruptors, (3) were in languages other than English, (4) did not present original findings (i.e., opinion pieces, letters to the editor) or (5) full information on the collected data was not available (i.e., conference abstracts).

To find the full breadth of research on the topic, the search was not restricted to year of publication. The studies examined in the review were collected through online databases, search engines and reference sections of published articles.

## 3. Endocrine Disruptor Compounds: Ambient Air Pollution, Persistent Organic Pollutants (POPs), Metals

Endocrine disruptor compounds (EDCs) comprise a range of substances that impede the endocrine system’s hormonal synthesis, metabolism, binding, transport, secretion and transport to target organs [17,18]. EDCs are found in the environment, food, manufactured goods (polychlorinated biphenyls), plastics (phthalates, bisphenol A (BPA]), production goods (fragrance compounds, organobromine flame retardants, fluorosurfactants), combustants (polychlorinated dibenzodioxins/furans, polyaromatic hydrocarbons), pesticides, herbicides and metals (arsenic, cadmium) [19].

EDCs alter homeostasis, reproductive systems and developmental processes through various mechanisms. The most studied pathway is the nuclear receptor pathway. EDCs can emulate and disturb the cellular activities of estrogens (ERs), androgens (ARs), progesterone (PR) and thyroid hormone (TH) by binding to their hormone receptors. The estrogen-like EDCs can also activate non-genomic nuclear signaling via GPR30, a G protein-coupled receptor for estrogen. BPA, for example, has a higher affinity for GPR30 than estradiol, inducing estrogenic effects. Other pathways involve non-nuclear steroid hormone receptors, non-steroid receptors (serotonin, dopamine and norepinephrine receptors), orphan receptors, aryl hydrocarbon receptors and chemical pathways involved in steroid synthesis and metabolism [17,19,20,21,22].

By exerting androgenic and estrogenic effects, EDC exposure is linked to adverse impacts on reproductive health. Maternal lead exposure raises the risk for spontaneous abortion, pre-term birth and smaller gestational size [23]. EDCs can also activate histone methyltransferase and alter histone methylation patterns of genes associated with prostate cancer via non-genomic signaling [22]. Evidence suggests that early puberty could be caused by exposure to EDCs. Premature breast development is associated with a higher concentration of phthalates, and sexual precocity is associated with higher levels of a dichlorodiphenyltrichloroethane (DDT) metabolite.

EDCs, such as BPA and phthalates, also impact adrenocortical function, impair the immune system and increase the risk for development of metabolic conditions such as diabetes and obesity [19,20]. In utero, exposure to polycyclic aromatic hydrocarbon (PAH) alters methylation and increases the activity of the primary regulator of adipogenesis, peroxisome proliferator-activated receptor-gamma (PPAR-γ) [19,20,22].

### 3.1. Ambient Air Pollution

According to the World Health Organization (WHO), air pollution is one of the greatest environmental threats to health. In 2016, 4.2 million deaths in cities and rural areas were estimated to be caused by air pollution. This threat significantly affects low- and middle-income countries, with 91% of the deaths found in areas such as South-East Asia and Western Pacific regions.

Particulate matter (PM) describes a mix of sulfates, ammonia, sodium chloride, black carbon, mineral dust and water suspended in the air released from industrial sites, wildfires, dust, agricultural activities and, importantly, on-road vehicles [24,25]. PM is used to denote the extent of air pollution as, relative to other pollutants, PMs have the most severe impact on health [24]. PMs of 10 microns or less (PM_10_) are inhaled and penetrate deep inside the lungs. Even more detrimental, PMs of 2.5 microns or less (PM_2.5_) can not only penetrate the lungs, but their small size allows them to enter the bloodstream. PMs have been shown to cause adverse health effects at any concentration, and controlling exposure has directly resulted in a reduction in morbidity and mortality [24].

Environmental factors altering epigenetics, such as through DNA methylation or histone modifications, have been postulated as a mechanism by which environmental exposure can contribute to the development of metabolic diseases. For example, LINE-1 methylation has been linked to obesity-related disorders alongside insulin resistance, type 2 diabetes mellitus and cardiovascular disease [26]. Some studies have shown an association between exposure to PM_2.5_ and DNA methylation of genes involved in coagulation, inflammation and immune response [26]. Evidence has demonstrated an association between PM_10_ exposure and DNA methylation of genes influencing cardiovascular disease, respiratory disease, inflammatory immune responses and oxidative stress [26].

Air pollution could also produce reactive oxygen species (ROS) that affect the nuclear methylation–demethylation circuit, leading to systemic and locus-specific epigenetic changes by directly modifying methylated CpG sites or by impacting the expression of enzymes. This, in turn, leads to metabolic dysregulation, such as dyslipidemia and glucose intolerance [26].

Ground-level ozone is another harmful air pollutant. It is formed through the chemical reactions of nitrogen oxides and volatile organic compounds emitted from cars, solvents and industrial products upon exposure to heat and sunlight. Ozone levels can reach harmful concentrations on warm sunny summer days. Evidence shows that ozone pollution is associated with DNA methylation and interferon gamma hypermethylation [26].

### 3.2. Persistent Organic Pollutants

**Figure 1:**
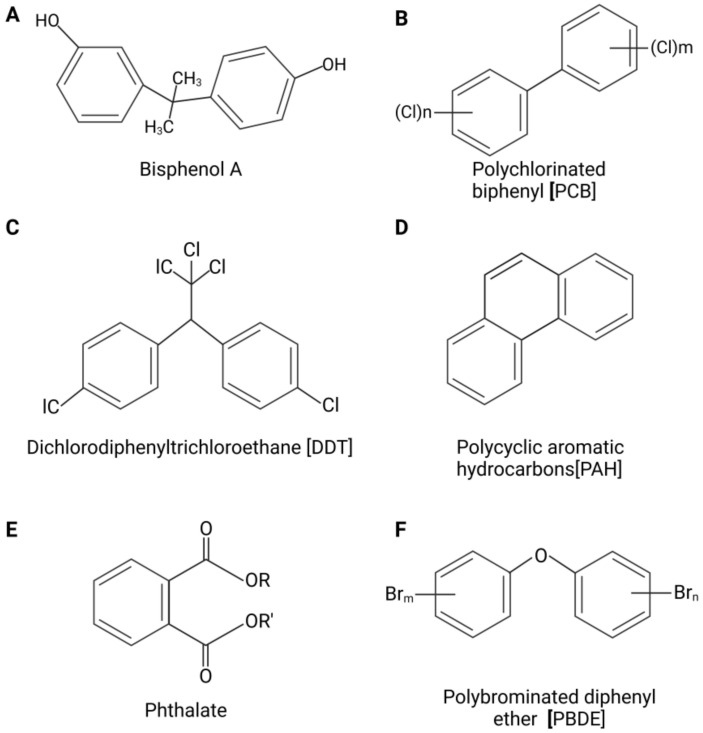
Chemical structures of persistent organic pollutants (POPs). Structures of bisphenol A (BPA) (**A**), polychlorinated biphenyl (PCB) (**B**), dichlorodiphenyltrichloroethane (DDT) (**C**), polycyclic aromatic hydrocarbons (PAHs) (**D**), phthalate (**E**) and polybrominated diphenyl ether (PBDE) (**F**).

Although changes are being implemented at local, national and international levels to reduce pollution, ban harmful chemicals and create a better environment for future generations, the impact on current individuals is still an ongoing issue. Some of the most potent forms of pollution are persistent organic pollutants (POPs) (Figure 1). POPs are industrial, manufactured chemicals found in the environment that exhibit a combination of physical and chemical properties that allow them to remain stable for long periods, thus accumulating in living organisms and the environment; they are toxic to humans and wildlife [27]. POPs are considered to be EDCs, as they can interfere with endocrine system homeostasis [20,28]. The sources and impacts of POPs on the endocrine system are summarized in Table 1. The Stockholm Convention was convened to ban or significantly reduce the release of 30 POPs to protect the environment, humans and wildlife. These POPs are divided into three annexes (Table 2) according to their level of restriction and production [29,30,31]. Annex A includes substances to eliminate, B to restrict and C to minimize and preferably eliminate unintentional release [32]. Despite these efforts, the long half-lives and range of transport, abundance in our foods and air, resistance to degradation and lipophilic nature of these chemicals have created a growing concern, and understanding their health impacts has become a priority [28,33]. Brominated and chlorinated POPs are lipophilic. Lipophilic POPs initially reside within lipid droplets that circulate in the bloodstream. These lipid droplets are then predominantly stored within adipocytes as an energy source [28,33]. This leads to an increased concentration of POPs in the adipose tissue [28,33].

#### 3.2.1. Polychlorinated Biphenyls

Polychlorinated biphenyls (PCBs) are manufactured organochlorine chemicals used in industrial production of a variety of items, particularly electrical products, that benefit from their low flammability [19,34]. The Stockholm Convention lists PCBs under Annexes A and C [30]. Their lipophilic nature, chemical stability and half-lives that span multiple decades explain their current abundance in the environment despite their ban in 1977 in the USA and their restriction in other parts of the world [28,43]. They are mainly inhaled or consumed in food due to their ability to pass up the food chain and be stored in adipose tissue. Two hundred and nine PCB compounds (congeners) have been identified and all share a similar structure: two connected phenolic rings bound to a maximum of ten chlorine molecules [43,44]. Each congener has its unique chlorine configuration that allows for a wide array of toxic effects on the endocrine system, the complexity of which has proven to be an obstacle to research efforts [19,44]. Dioxin-like PCBs are considered the most toxic and antiestrogenic. They bind to the aryl hydrocarbon receptor to induce cytochrome P450 CYP1A. Non-dioxin-like PCBs act on both the CYP2B enzyme and CYP1A and have estrogenic or antiandrogenic properties via acting upon steroid hormone receptors. PCBs have wide-ranging effects through their existence as a blend of multiple compounds and their ability to individually impact various pathways [44]. Non-dioxin-like PCBs 170 and 187 have been correlated with insulin resistance [45].

#### 3.2.2. Pesticides

Pesticides are widely used in agricultural settings to preserve crops, household items and even to eliminate disease vectors for the enhancement of public health safety [46]. Pesticides can accumulate in the body as they resist chemical and biological degradation. They can bind to the soil to remain in the environment or even volatize and shift to neighboring areas to be inhaled or consumed in the diet [35]. Of the twelve initial chemicals mentioned in the Stockholm Convention, nine are pesticides. Organochlorine pesticides (Table 3), such as dichlorodiphenyltrichloroethane (DDT), lindane and chlordane, are chlorinated hydrocarbons that were extensively used from the 1940s to the 1960s. Chlorinated hydrocarbons are lipophilic and are still prevalent in the environment despite subsequently being tightly controlled and limited. Organophosphate pesticides (Table 3) are the most frequently used insecticides. They kill pests by damaging acetylcholinesterase, an enzyme critical to nerve signals. They are rapidly broken down to their metabolites, thus not accumulating in the environment. Long-term exposure through direct contact, ingestion or inhalation can cause neuropathic symptoms such as weakness and numbness [47].

Despite their prevalence in the environment, epidemiologic data showcasing their impact on our health are limited. However, a large comprehensive systematic review has found an association between organochloride pesticides and an increased risk of type 2 diabetes mellitus. The pathogenesis has yet to be elucidated [48].

#### 3.2.3. Polycyclic Aromatic Hydrocarbons (PAHs)

Another common group of POPs is the polycyclic aromatic hydrocarbons (PAHs). PAHs are organic compounds with two or more fused aromatic rings. They are released into the atmosphere through tobacco smoke, industrial processes, gasoline, coal and food preparation. They are also naturally formed from burning biomass materials [19,38]. Once released into the environment, they are inhaled or consumed in the diet. PAH concentration is notably elevated in developing countries and urban settings [19]. Toxicity resulting from exposure to high levels of PAHs is theorized to occur through two mechanisms depending upon the specific PAHs: either reactive diol epoxide intermediate formation covalently binding to DNA through CYP450 catalyzation or reactive oxygen species (ROS) production causing oxidative stress and inflammatory responses. PAHs can lead to abnormal glycation of proteins and lipids as advanced glycation end products (AGEs) whether, for example, through the Maillard reaction to increase the preservation of food or through the release of tobacco smoke [49]. In the body, these AGEs can disrupt receptors and increase the risk of developing obesity in utero, and exposure to polycyclic aromatic hydrocarbon (PAH) alters methylation and increases the activity of the primary regulator of adipogenesis, peroxisome proliferator-activated receptor-gamma [22].

#### 3.2.4. Bisphenol A

Bisphenol A (BPA) is an organic POP used to produce plastic products and epoxy resins [19,35]. More than 8 billion pounds are produced annually. Approximately 100 tons are released into the atmosphere, with the hypothesized primary source coming from burning plastic [19]. BPA is a well-known EDC. Long-term exposure to BPA promotes adipogenesis, lipid accumulation and proinflammatory cytokine production while reducing insulin sensitivity [19,35]. The primary mechanism of BPA action is through disruption of critical regulators in these processes. Animal studies have linked a high BPA exposure in utero to an imbalance in proinflammatory and anti-inflammatory immune responses and an increase in proinflammatory Th1 response, which leads to negative consequences such as production of antigen-specific antibodies and development of food intolerance [50]. Additionally, in vitro exposure of human subcutaneous adipose tissue to BPA increased levels of interleukin 6 (IL-6) and tumor necrosis factor-α (TNF-α) while decreasing adiponectin, an adipokine that improves insulin sensitivity [50]. Equally, in 3T3-L1 cells (a mouse adipocyte cell line), BPA exposure increased cytokine levels and insulin resistance in the c-Jun N-terminal kinase (JNK) and nuclear factor kappa B (NF-κB) pathways [50]. However, further in vivo experiments are needed to clearly delineate the effects of BPA.

#### 3.2.5. Phthalates

Phthalates are regularly used in the production of plastics to increase their flexibility and strength [19]. As well as their use as plasticizers, they can be used as matrices and fillers [40]. The lighter, more volatile phthalates, dimethyl phthalate, diethyl phthalate and dibutyl phthalate, are found at higher concentrations in the air versus the heavier, less volatile phthalates, such as bis(2-ethylhexyl) phthalate (DEHP) and butyl benzyl phthalate (BBP), that are more widely found in household dust [19]. DEHP is also commonly found in medical devices. Standard routes of exposure are through inhalation, ingestion and application of cosmetic products [40]. Phthalates are measured in urine, as they have short half-lives and renal excretion is the main clearance method. Phthalates, as with most POPs, are EDCs with strong antiandrogenic and weak estrogenic properties [51]. Phthalate exposure is believed to be linked to obesity and insulin resistance. Indeed, phthalates bind to PPAR-γ, causing an increase in adipocyte production and insulin resistance [52].

#### 3.2.6. Polybrominated Diphenyl Ethers

Polybrominated diphenyl ethers (PBDEs) are organobromines composed of 2 halogenated rings bound to up to 10 bromine atoms, forming 209 different congeners, similar to PCBs [19]. They are the main component of flame retardants and are sold in three different mixtures according to their degree of bromination. All three varieties have been progressively withdrawn from commercial use in the USA due to their toxicity and bioaccumulative properties [41]. However, exposure through ingestion, inhalation and dermal absorption, mainly from dust, has not ceased due to their decade-long half-life. Unlike PCBs and chlorinated components, whose levels have decreased in the last 30 years, PBDE levels have increased appreciably. Levels in human tissues are higher in North America than in Europe and Japan [41]. Epidemiologic studies have demonstrated an association between PBDE exposure and metabolic disease. A hypothesis requiring further investigation postulates that PBDE mediates a decrease in a gut microbiota-derived metabolite of tryptophan (3-IPA), disrupting glucose and insulin signaling. Furthermore, as 3-IPA suppresses inflammatory genes by antagonizing aryl hydrocarbon receptor functions, its reduction could contribute to diabetes [53].

### 3.3. Metals

Arsenic (As) is a toxic, naturally occurring metalloid abundant in the environment. Its primary route of entry is through ingestion, inhalation and, to a lesser degree, absorption through cutaneous contact [54]. Arsenic is found in the environment due to both artificial and natural processes. It is released naturally through groundwater, mineral ore and geothermal processes [55]. It is found in foods, particularly shellfish. Artificially, it is found in glass manufacturing, herbicides and pesticides, and its use is widespread in the electronics industry. In 2003, China, Chile and Peru were the top three producers of arsenic whilst the United States was its most significant consumer. Today, the USA’s leading form of arsenic production is in the manufacturing of wood preservatives [55]. Arsenic contaminates water, making it one of the top environmental health threats worldwide. The metalloid plays a role in the development of diabetes through various mechanisms. It modifies gene transcription, affecting insulin signal transduction, adipocyte differentiation and insulin sensitivity [56]. In rat studies, administering arsenite to insulin-sensitive cells decreased glucose uptake [56]. In addition, when arsenite was added to pancreatic β-cells, reduced insulin secretion and synthesis were found [56]. Unfortunately, due to the high discrepancies in prevalence between and even within countries, it is challenging to find irrevocable evidence of causation between chronic arsenic exposure and disease [57].

Cadmium (Cd) is a toxic metal found naturally in the Earth’s crust and is emitted into the air through volcanic eruptions and forest fires. It is used artificially to form batteries, plastics, pigments and cigarettes. Consuming contaminated food and water is the main source of exposure [58]. Cd exposure is believed to affect the incidence of obesity by acting on PPAR-γ, causing hyperplasia and hypertrophy of adipose tissues, disrupting endocrine homeostasis, altering appetite and satiety regulation and affecting insulin sensitivity [59].

## 4. The Molecular and Cellular Mechanisms of Diabetes and Obesity

### 4.1. Molecular Mechanisms

Insulin resistance is a hallmark feature of both obesity and type 2 diabetes. In insulin resistance, cells in the body become resistant to the actions of insulin, leading to a decrease in glucose uptake and metabolism. This results in increased blood glucose levels, which can lead to diabetes [1,2].

Obesity is associated with chronic low-grade inflammation in adipose tissue, which can lead to the release of cytokines and adipokines, such as TNF-alpha and leptin, which contribute to the development of insulin resistance [1,2,3].

Dyslipidemia, characterized by elevated levels of triglycerides and low-density lipoprotein (LDL) cholesterol and decreased levels of high-density lipoprotein (HDL) cholesterol, is frequently observed in obesity and diabetes [1,2].

Oxidative stress, which occurs due to an imbalance between reactive oxygen species (ROS) and antioxidants, is associated with insulin resistance, inflammation and cellular damage [1,2].

Transcription factors are proteins that bind to specific DNA sequences and regulate the expression of genes and these play a role in the development of obesity and diabetes. One key example is peroxisome proliferator-activated receptor-gamma (PPAR-γ). PPAR-γ is highly expressed in adipose tissue and its activation leads to an increase in adipocyte differentiation and fat storage. PPAR-γ also plays a role in insulin sensitivity by increasing glucose uptake in adipose tissue and skeletal muscle [8,9,10].

Another key transcription factor involved in obesity and diabetes development is sterol regulatory element-binding protein-1c (SREBP-1c). SREBP-1c is involved in the regulation of lipogenesis. In obesity, SREBP-1c is overexpressed, leading to an increase in fat storage and insulin resistance [1,2].

Enzymes also play a critical role in the development of obesity and diabetes, one example being hormone-sensitive lipase (HSL). HSL is responsible for breaking down stored fat molecules in adipose tissue. In insulin-resistant individuals, HSL activity is decreased, leading to an increase in fat storage and obesity [1,2,8,9].

Another key enzyme involved in diabetes development is glucokinase. Glucokinase is responsible for the phosphorylation of glucose in pancreatic β-cells, which is the first step in insulin secretion. In type 2 diabetes, glucokinase activity is decreased, leading to a decrease in insulin secretion and hyperglycemia [1,2,11].

### 4.2. Cellular Mechanisms

Cellular mechanisms also contribute to the development of obesity and diabetes. Adipose tissue is a major site of energy storage, and its dysfunction is a key feature of obesity. In obesity, adipose tissue expands and becomes inflamed, leading to a decrease in adiponectin secretion and an increase in proinflammatory cytokine release. This inflammation leads to insulin resistance and further exacerbates obesity [1,2,3].

Skeletal muscle is a major site of glucose uptake and utilization. In insulin-resistant individuals, skeletal muscle becomes less responsive to insulin, leading to decreased glucose uptake and metabolism [1,2,3].

In obesity and diabetes, the liver becomes insulin resistant, leading to increased glucose production and release into the bloodstream [1,2,8].

In diabetes, pancreatic β-cell dysfunction is a critical feature. β-cells become dysfunctional and fail to secrete adequate amounts of insulin, leading to hyperglycemia. This dysfunction is due to the chronic exposure of β-cells to high levels of glucose and fatty acids, which provokes β-cell apoptosis [1,8,11].

In summary, molecular and cellular mechanisms play critical roles in the development of obesity and diabetes. Transcription factors and enzymes are among the molecular mechanisms involved, while dysfunctions of adipose tissue, pancreatic β-cells, liver and skeletal muscle are among the cellular mechanisms involved.

## 5. The Impact of Pollution on Obesity

### 5.1. The Development of Obesity

Obesity is an ongoing pandemic affecting many nations across the globe, with an exponential growth in prevalence over recent years. Obesity has many risk factors associated with its development, including genetic predisposition and environmental impacts. Three main mechanisms have been proposed for the link between pollution and obesity: physical inactivity, oxidative stress caused by pollutants and epigenetic modifications [60].

**Figure 2:**
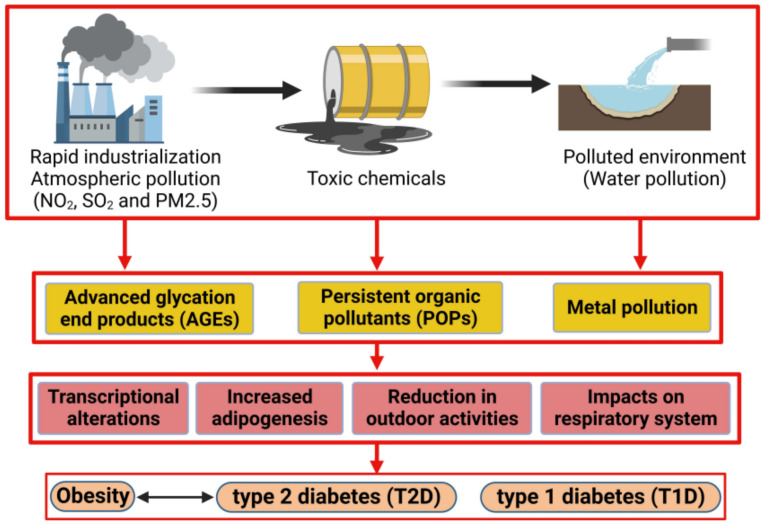
Effect of environmental pollution in developing metabolic disorders: rapid industrialization, toxic chemicals and a polluted environment generate a massive number of endocrine disruptor compounds (EDCs), for example, advanced glycation end products (AGEs), persistent organic pollutants (POPs) and metal pollutants, that affect the normal physiological functions in the human body. Disruption of cellular and physiological activities in response to EDCs increases the risk for development of obesity and diabetes.

Rodent studies indicate that ambient air pollution behaves as an obesogenic factor, disrupting typical adipose function [60]. These ambient air pollutants alter specific metabolic functions, such as storage and breakdown of fats, leading to an increase in weight [61]. Although not conclusive, especially in humans, there is a growing field of research surrounding the impact of pollution due to the growth of cities, the increased density of urban areas and the overall global industrial shift over the years likely contributing to the alarming prevalence of metabolic disorders such as obesity and diabetes (Figure 2).

### 5.2. Advanced Glycation End Product (AGE) Impact on Obesity Prevalence

**Figure 3:**
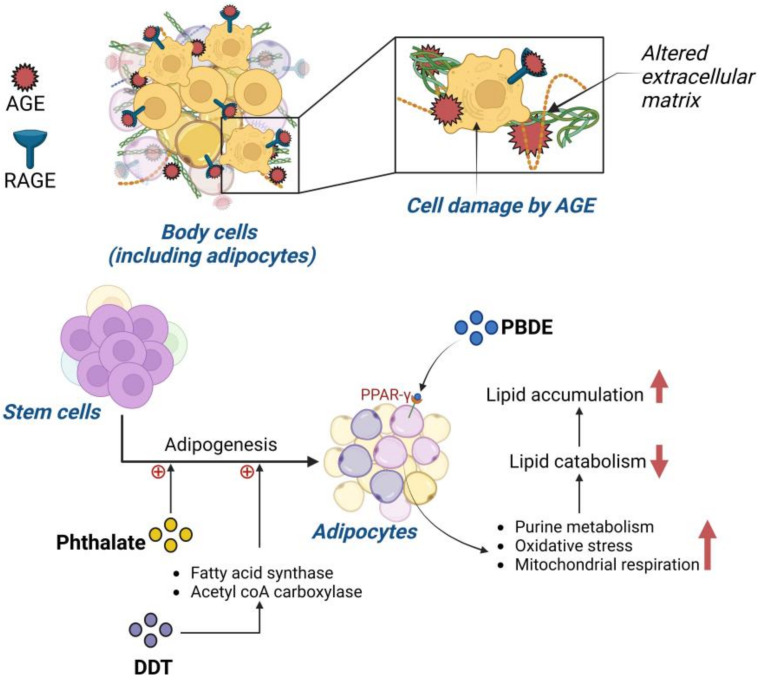
Mechanism of persistent organic pollutant (POP)-induced obesity: (**upper panel**) illustration showing interaction of advanced glycation end products (AGEs) and receptor of AGE (RAGE) in cells (including adipocytes). AGE binds to RAGE expressed in cells as well as in the extracellular matrix (ECM), altering the structure of ECM and causing cellular damage. (**Lower panel**) diagram illustrating the effect of phthalate, DDT and PBDE in adipogenesis. Phthalate and DDT induce stem cells to differentiate to adipocytes. PBDE binds to the peroxisome proliferator-activated receptor-gamma (PPAR-γ) and enhances lipid accumulation. An upward red arrow indicates an increase and a downward red arrow indicates a decrease in the rate of the specified metabolic pathway.

As many nations continue to industrialize, fast food and processed foods have become increasingly prevalent. This has drastically altered human dietary intake. Through the processing of foods, advanced glycation end products (AGEs) have become an important aspect of food consumption [62,63]. AGEs are produced when proteins, lipids or nucleic acids are glycated through the unnatural processing of foods that involves various chemicals used for increasing product storage life [62,63]. Common AGEs are pentosidine and N-[carboxymethyl]-lysine (CML), caused by the increased presence of glucose [64]. AGEs function by forming a link between the membrane of molecules and specific receptors known as receptors for advanced glycation end products (RAGEs) [65]. The ability of AGEs to bind to the basement membrane of the extracellular matrix alters cell function and structure [65]. The recruitment of RAGEs further changes the cell’s ability to function [65] (Figure 3). Through their binding, AGEs can alter collagen, laminin and vitronectin [65]. These three proteins are integral parts of vascular homeostasis; thus, AGEs have an impact microscopically and macroscopically [65]. The increase in processed foods has paralleled the timeline and growth of the obesity pandemic, and research has investigated whether there is a correlation between these AGEs and obesity. A study of 4245 participants found that individuals who consumed higher amounts of AGEs had increased abdominal obesity [62]. Rather than the quantity of food or the caloric intake, it was the consumption of AGEs that caused the increased adiposity [62].

### 5.3. Impact of Persistent Organic Pollutants on Obesity

Bisphenol A has a link to obesity and body mass index (BMI), mainly through the alteration of adipogenesis regulators [35,66]. BPA leads to the increase in mRNA for 11b-hydroxysteroid dehydrogenase type 1 (HSD1), an enzyme affecting adipogenesis regulation [67]. In addition, bisphenol A binds to glucocorticoid receptors activating 11b-HSD1, accelerating the process of adipogenesis [67].

Due to the negative impact of BPA, other components used in plastics were evaluated for their effects on the human body. One of those components was phthalate and another was POP. Urinary content analysis found that increased phthalate metabolites were positively correlated to three factors: obesity, triglycerides and increased blood pressure [35,68]. A remarkable yet detrimental feature of phthalate function is the ability to promote adipogenesis from stem cells rather than osteoblast genesis, causing increased fat build-up rather than required bone quality improvement [35,69] (Figure 3). Phthalates, specifically mono-2ethylhexyl-phthalate, bind to PPAR-γ, a receptor that influences adipocyte differentiation [59]. PPAR-γ is a key regulatory receptor that influences adipogenesis through a transcriptional cascade [59]. Many genes associated with adipogenesis are activated and upregulated once PPAR-γ is activated. This receptor also contributes to adipocyte insulin sensitization, disrupting blood glucose homeostasis [70]. POPs found in plastic similarly impact human physiology and are worrisome as POP-infused plastics are widely used for storing water, food and other commonly used objects.

Another widely used POP is DDT. A study conducted on 298 individuals in Spain found that lipid DDT levels positively correlated with BMI. A more recent study on 429 adults found that DDT and its metabolites alter the fasted glucose state, further contributing to obesity development [28,35,71]. Laboratory investigations confirmed the potency of DDT and its adipogenicity effects; DDT was found to promote adipogenesis by increasing the presence of fatty acid synthase, acetyl-CoA carboxylase and lipid accumulation [28,35,72]. The main mechanism for these increases relates to DDT and its subsequent upregulation of PPAR-γ, leading to the transcriptional cascade influencing adipogenesis [22,28]. DDT has also been seen to have an influence on insulin resistance of adipocytes [22,28], thus leading to further dysregulation of lipid and carbohydrate metabolism, adipose tissue accumulation and metabolic disruption [22,28] (Figure 3).

A major concern is that the accumulation of PBDEs in adipose tissue closely correlates with insulin resistance and therefore the development of diabetes [28,35,73,74]. Laboratory studies have shown that obesogenic effects are correlated to the presence of PBDEs, specifically in the promotion of adipogenesis through lipid level alteration [28,35,75]. PBDE accumulation was found to have links to certain obesity biomarkers such as leptin and adiponectin, as well as increased PPAR-γ expression [35]. However, another hypothesized obesogenic mechanism for PBDE is through the activation of purine metabolism, with an increase in oxidative stress and increased mitochondrial respiration [35]. These three factors were seen to decrease lipid catabolism, allowing lipids to accumulate within adipose tissue [35].

Perfluorinated compounds, another form of POP, have been shown to be inducers of adipogenesis [28,35]. These compounds, like PBDEs, also play a role in the insulin signaling pathway, which raises concerns about their promotion of other metabolic disorders [28,35,76]. Perfluorinated compounds influence insulin-stimulated glucose uptake by an increase in both glucose transporter type 4 (GLUT4) transportation and insulin receptor substrate-1 (IRS1) [35]. PCBs have already been banned in many places due to their ability to imitate key hormones of the endocrine system [28,35,76]. A linear relationship between PCBs and waist circumference has been documented [28,35,77]. Lipid metabolism is another target of these chemicals, due to their accumulation in adipose tissue [28,35,77]. PCBs were found to accumulate specifically within lipid droplets. They have a role in regulating PPAR-γ as well as CCAAT/enhancer-binding protein-alpha, two receptors that are adipogenic factors [35]. Perfluorinated compounds influence insulin-stimulated glucose uptake by an increase in both GLUT4 transportation and IRS1 [35].

Another group of POPs in common use are the polycyclic aromatic hydrocarbons (PAHs). Investigation of 3189 urine samples from those exposed to PAHs found a positive association between body mass index (BMI), obesity and PAH concentration [28,35,78]. PAHs impair adipose tissue lipolysis, as well as affect PPAR-γ [78]. Reducing exposure to POPs, particularly in infants and children, is important in efforts to reduce the prevalence of obesity and other metabolic disorders [28,35,78].

### 5.4. Pollution and the Impact on Childhood Obesity

Several disorders have roots in the pre-natal environment as, when the pre-natal environment is disrupted, a higher prevalence of certain disorders occurs in later life. In studies researching the impact of pollutants on the pre-natal environment, pre-natal exposure to various types of pollutants can promote childhood obesity [79]. In three separate studies using Project Viva (a Boston-area pre-birth cohort including 1649 children), mothers living in high traffic density areas had children with a higher likelihood of developing obesity between the ages of six months and 10 years of age [79,80,81]. Smoking was another factor studied to determine the impact on the pre-natal environment and the potential risks it could impose on the future child. In two studies, one with 21,063 mother–child pairs and one with 13,188 singleton children, it was demonstrated that smoking or high exposure to secondhand smoking (tobacco) led to overweight children through evaluation of BMI at the ages of three and seven years [82,83]. A positive association between pollutants in the pre-natal environment and childhood obesity was found with polycyclic aromatic hydrocarbons (PAHs). Children with increased pre-natal exposure to PAH were seen to have an increased prevalence of obesity from 20.6% at age 5 to 33% at age 11 in comparison to those with no or minimal PAH exposure [84,85]. Post-natal early exposure to pollution was also shown to be detrimental to long-term metabolic health. When evaluating children at an average age of 6.6 years old, it was found that those living in high-density traffic areas had a 2.6 kg/m^2^ increase in their BMI compared to those in less dense areas at three-year follow up [86]. Whether due to pollution from vehicle exhausts or a pollution-induced reduction in physical activity, these children were put at metabolic risk from living near dense traffic [86]. A study in mice demonstrated that early life exposure to particulate matter could lead to many risk factors associated with obesity such as insulin resistance and accumulation of macrophages within adipose tissue [61]. A reduction in early life pollution exposure is important for reducing the prevalence of metabolic disorders occurring in later life.

Obesity’s prevalence continues its dramatic increase worldwide and it is critical to find a way to curb this epidemic. Lifestyle changes are key to reversing obesity, but these changes are challenging for patients to maintain. Nutritionally healthy meals and increased physical activity are the cornerstones of lifestyle modification. Exercise can “flush out” POPs, due to the progressive loss of adipose tissue, the major storage site of POPs [87]. Exercise is not only beneficial for weight loss and improving overall health, but also for reducing the impact of pollutants on the body through the mechanism noted above [87]. Obesity itself is a risk factor for the development of type 2 diabetes (T2D) and the continuing increase in obesity is reflected in the increasing prevalence of T2D.

## 6. Impact of Pollution on Type 2 Diabetes Mellitus (T2D)

### 6.1. The Development of T2D

Characteristics of T2D are hyperglycemia, increased peripheral insulin resistance and increased lipid oxidation. Chronic hyperglycemia is due to an inability of the body to consistently break down the glucose required to keep up with energy requirements because of peripheral insulin resistance. Peripheral insulin resistance results in a consequent reduction in the uptake of glucose by insulin-dependent glucose transporters (GLUT4), resulting in an increase in circulating glucose, and subsequent lipid oxidation due to impaired glucose intake [88].

While a variety of research indicates a direct link between pollutants and the onset of diabetes in human populations, several studies have deduced the association of pollutants with risk factors such as hyperglycemia, peripheral insulin resistance, hepatotoxicity and metabolic disruption, that are frequent forerunners of T2D.

### 6.2. The Role of Metals in Inducing Type 2 Diabetes Mellitus

**Figure 4:**
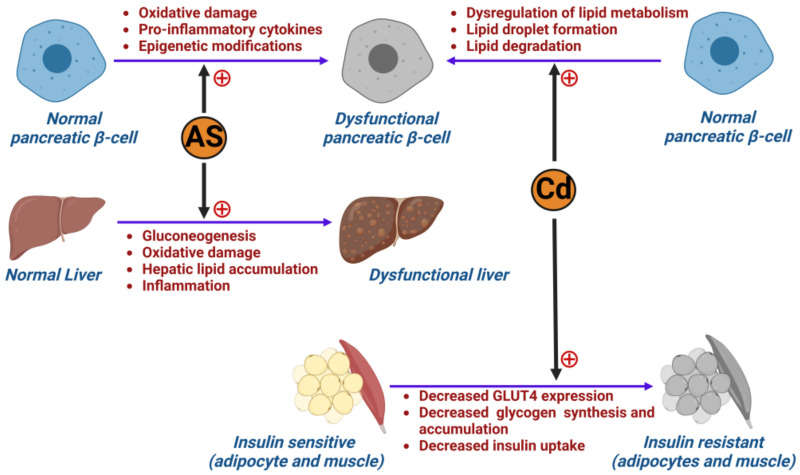
Effect of metal pollution in developing diabetes: illustration showing how arsenic (As) and cadmium (Cd) cause dysfunction in pancreatic β-cells, liver and insulin-sensitive tissues (adipocytes and muscle), leading to the development of diabetes.

The heavy metal arsenic (As) has been posited to have negative pancreatic and hepatic effects (Figure 4). A review identified that arsenic plays a role in inducing diabetes by altering tumor necrosis factor-α (TNF-α), GLUT4 and mitogen-activated protein kinase (MAPK) [88]. In a rodent study conducted by Liu et al., the control group of mice was shown to be affected by inorganic arsenic through pancreatic β-cell dysfunction with increased gluconeogenesis and oxidative damage in the liver [89]. The group of diabetic mice within the same study were shown to have even worse glucose tolerance. Ongoing arsenic exposure was also found to impact liver function as it induces hepatic lipid accumulation [90] and triggers expression of genes involved in the onset of inflammation within the liver [91]. Hyperglycemia induced by impaired liver function instigates oxidative damage and decreases glycogenesis. As such, arsenic exposure can play a role in the development of ROS through the inhibition of mitochondrial function, onset of lipid peroxidation and further mitochondrial damage [92]. Oxidative stress on the pancreas is also triggered by accumulation of arsenic within the body [93,94], resulting in diminished insulin sensitivity and β-cell apoptosis [94]. Arsenic exposure in a rodent study conducted by Khan et al. also resulted in alterations in the methylation pattern of the glucose transporter 2 gene (GLUT2), and changes to the insulin gene (INS, coding for insulin) and pancreatic and duodenal homeobox-1 (Pdx1, that regulates pancreatic cell proliferation) [90]. By altering the methylation pattern of GLUT2, a subsequent increase in GLUT2 expression was induced [93]. However, after exposure to sodium arsenite, INS and Pdx1 expression was decreased. It should also be noted that arsenic has implications in affecting fetuses through transplacental exposure. Therefore, not only were mothers seen to be negatively impacted by exposure, but their offspring as well, with future metabolic dysfunction after birth as arsenic accumulates in the liver of neonates [95]. Thus, these compounding factors would induce insulin resistance and further risk for development of T2D [91,93].

As mentioned above, arsenic is not the sole metal found to have toxic effects over long-term exposure. The persistence of human activities, such as agriculture and industrialization practices, lifestyle and diet, has caused exposure to cadmium (Cd) [94,96] (Figure 4). Levels of exposure vary widely from country to country, with countries in Eastern Asia experiencing significantly higher exposure versus Western countries (USA and European Union) as reported by the WHO [94,97]. The exact mechanism of diabetogenesis resulting from cadmium exposure has not yet been fully elucidated. However, several studies suggest that it plays a prominent role in affecting the pancreas and the production of insulin. In a mouse study conducted by Hong et al., Cd application to MIN6 cells (a mouse insulinoma-derived cell line that serves as a model of pancreatic β-cell function) led to dysfunction in lipid metabolism and was linked to pancreatic β-cell dysfunction and death [97]. The study concluded that exposure could lead to accumulated lipids in pancreatic β-cells and cause dyslipidemia in vivo. This was demonstrated by a distinct increase in lipid droplet formation in pancreatic β-cells, with decreased lipid degradation. As Cd accumulates in the β-cells, it decreases their ability to release insulin and therefore results in increased circulating blood glucose levels [98]. Cd exposure has also reportedly caused pancreatic β-cell dysfunction via inducing hyperglycemia and affecting the lipid oxidation of pancreatic cells [96]. Some studies have also indicated that the risk of developing T2D in Cd-exposed patients is increased in those under the age of 50 via increasing the likelihood for peripheral insulin resistance [99]. This was demonstrated in a rodent study [100] where, following administration of CdCl2, expression of GLUT4 in skeletal muscle and adipocytes was decreased, resulting in further decreased glycogen synthesis and insulin-dependent uptake of glucose [100,101]. In a meta-analysis comparing high to low amounts of exposure, higher exposure translated into higher risk for developing T2D (odds ratio 1.38, 95% confidence interval 1.12–1.71) [102]. Whilst Tinkov et al. conducted a meta-analysis that found strong associations between Cd exposure and the development of pre-diabetes (*p* < 0.01) [102], other studies have shown no association between Cd and diabetes (*p* = 0.347) [103]. These conflicting findings may be due to the differing exposure markers (urine, nails, hair, HbA1c testing) used in the studies to determine Cd exposure. However, laboratory studies included in this meta-analysis concluded that Cd exposure heightened insulin resistance and, therefore, augments T2D incidence [104,105]. To support the limited studies available, more research is needed to determine whether there is a definitive link between Cd exposure and T2D.

### 6.3. Air Pollution and Its Impact on Developing Type 2 Diabetes (T2D)

The pathogenesis of T2D is directly associated with insulin resistance, which can be triggered by systemic inflammation and oxidative stress and has been postulated to be triggered by exposure to air pollution. In a mouse study conducted by Sun et al., 24 weeks of exposure to PM_2.5_ induced insulin resistance, increased visceral adipose tissue and caused immune response dysregulation [106]. The Study on the Influence of Air Pollution on Lung Function, Inflammation and Aging (SALIA) was a cohort study of women aged 54–55 years old who lived in some of Germany’s most densely polluted districts [107,108]. Consecutive cross-sectional surveys were conducted between 1985 to 1994 to identify the effects of industrial and traffic-related pollution exposure [107,108]. A follow up investigation conducted by Krämer et al. on the SALIA cohort included 1775 participants from the prior study to whom they administered self-reported questionnaires. In doing so, they were able to identify an association between NO_2_ exposure from traffic pollution and T2D with an incidence of 10.5% onset T2D cases [109]. Additionally, a cross-sectional study of 374 participants aged 10–18 years old conducted in Iran indicated that, with ongoing increased exposure to traffic-based and industrial air pollution (resulting in increased PM_10_ exposure), homoeostasis model assessment model-insulin resistance (HOMA-IR) results were elevated and concluded there to be a direct association of air pollution with insulin resistance [109,110]. While many studies have identified a positive association with pollution risk factors and development of T2D, there is a paucity of mechanistic information as to how air pollution causes T2D.

## 7. The Impact of Pollution on Type 1 Diabetes Mellitus (T1D)

Type 1 diabetes mellitus (T1D) is characterized by autoimmune destruction of the pancreatic β-islet cells, the cells responsible for the production of insulin. The underlying cause of this autoimmunity is still not fully understood although certain genetic predispositions to T1D development are known. Certain factors that work in concert with the underlying genetic predisposition to promote disease onset include epigenetic modifications, pancreatic β-cell destruction and inflammation [107,108]. Epigenetic alterations, transcriptional alterations and the impact of gene expression are all areas where environmental pollutants can impact upon a genetic predisposition [107,108]. Research is actively being conducted to evaluate how pollution impacts metabolic disorders, but more research directed at pollution and T1D specifically is needed.

### 7.1. Impact of Pollution on Pancreatic β-Islet Cells

Pollutants seem to have an affinity for pancreatic β-islet cells both during early development and once the organ is fully formed [111]. In a study investigating the impact of dioxin on pancreatic β-cells, it was reported that β-cells were unable to secrete insulin when subjected to dioxin [111]. The cause was determined to be an alteration in expression of certain genes required for the maintenance of insulin levels and β-cell function that included glucokinase (Gck), B-cell lymphoma-extra-large (Bcl-xL), Pdx1, forkhead box O1 (FoxO1), inositol-requiring enzyme 1 (IRE1), GLUT2 and musculoaponeurotic fibrosarcoma oncogene family A (MafA) [111].

Research into the impact of cadmium, a metal pollutant, on the pancreas has been minimal to date [97]. When testing the impact of cadmium on pancreatic β-cells of mice, a multifactorial effect was seen [97]. Cadmium caused increased lipid accumulation in β-cells due to upregulation of cellular lipogenesis [97]. Cadmium also provoked a proinflammatory response leading to increased production of proinflammatory cytokines, a risk factor for diabetes development [97]. These key aspects lead to β-islet cell dysfunction or death whether in vitro or in vivo, further accelerating the potential for diabetes development.

### 7.2. Impact of Pollution on Childhood Type 1 Diabetes Mellitus

Although the onset of type 1 diabetes (T1D) can occur throughout life, it frequently occurs in children. Pollution studies have primarily investigated the impact of toxic airborne particles on T1D in children. In a study analyzing data from 19 European countries, there was a positive association between pollution exposure and the onset of T1D for individuals in 18 of those countries [112]. The target demographic for this study was individuals diagnosed with T1D from the age of 0–15 years [112]. Emission information was gathered from the European Environmental Agency to evaluate several toxic emissions because it is recognized that individuals experience a plethora of toxins within the atmosphere concurrently rather than in isolation [112]. Findings from this article are important as it highlights the relationship between pollution and T1D and the potential that pollution reduction could lower the prevalence of this disorder.

To further emphasize the early impact of pollution on children and T1D, a study compared ozone exposure in T1D and healthy children. Children with T1D had a higher pre-diagnosis exposure to ozone than healthy children [113]. However, the key concept highlighted by this study was that certain pollutants increase the likelihood of T1D onset before the age of 5 years [113]. Sulfur dioxide (SO_2_) exposure was associated with a later onset of diabetes versus those with early-onset disease [113]. Therefore, ozone has been hypothesized to be a pollutant linked to the increased earlier incidence of diabetes amongst children [113]. This is of great concern, as ozone levels and other atmospheric pollutants have risen in recent years, despite the current ongoing efforts to curb atmospheric pollution levels.

Atmospheric pollution is not the only form of pollution affecting younger populations. The presence of persistent organic pollutants (POPs) is another area of concern for T1D development. When studying 442 youths for the impact of POPs, with a focus on their impact on β-cells, a clear relationship was found [114]. Initially, the study investigated the odds ratio of T1D development in the presence of POPs and found that those subjected to dichlorodiphenyldichloroethylene (DDE), a metabolite of the POP DDT, had an increased chance of T1D development [114]. When POPs were introduced to isolated β-cells, after just two days the cells were non-viable [114]. Additionally, the presence of POPs shut down insulin production due to alterations in Ins1 and Ins2 mRNA expression [114].

When examining phthalate exposure in mothers, the expression of Pdx1, a gene required for β-cell production, was downregulated [115,116]. Pdx1 not only has a role in β-cell production, but also in mitochondrial function [115,116]. During pancreas development, large quantities of energy are required for β-cell development and, with reduced functionality of mitochondria, increased production of ROS occurs [115,116]. Oxidative stress damages mitochondrial DNA and, later in life, is associated with adult-onset diabetes [115,116]. The presence of this POP found in plastic not only causes a reduction in β-cell production but also has a detrimental impact on mitochondrial function. Pre-natal and childhood periods are crucial for the growth and development of a healthy pancreas [117,118]; environmental impacts before and after birth can be detrimental in both the short and long term.

The work presented here shows that there are risk factors other than genetics associated with the onset and progression of type 1 diabetes. More work needs to be carried out to evaluate how to reduce the effect of these pollutants and reduce their impact on future generations. Pollutants have a clear and susceptible target in pancreatic β-cells by targeting critical genes. Despite T2D being more conventionally recognized as having environmental risk factors, more work must be conducted to understand how pollution causes an increase in T1D incidence as well. Though limited, the available literature has highlighted the relationship between POPs and ambient air pollution and T1D.

## 8. Impact of Pollution on Gestational Diabetes (GDM)

### 8.1. The Development of GDM

During pregnancy, the placenta secretes hormones to sustain the fetus, impacting insulin resistance around the 20th to 24th week of gestation. In normal conditions, the pancreas counteracts this phenomenon by increasing the production of insulin through β-cell expansion [119,120,121]. However, when underlying β-cell dysfunction and impaired insulin secretion are present, the mother cannot maintain glucose homeostasis [119,122] and gestational diabetes mellitus (GDM) results. GDM has significant short- and long-term consequences on the mother’s and offspring’s health [122]. The fetus’ primary energy source is glucose, thus making the placenta very sensitive to the prevailing hyperglycemia of GDM [123]. This intrauterine exposure increases the probability of macrosomia, congenital disabilities, stillbirth, developmental delays, pre-diabetes and obesity [119,124,125,126]. When uncontrolled, GDM causes high blood pressure, increased cardiometabolic diseases and a greater incidence of T2D development later in life despite the glucose intolerance normally regularizing in the mother after delivery [119,127]. Indeed, according to the CDC, around 50% of people with GDM develop T2D, thus contributing to the worldwide epidemic. GDM is now one of the most common metabolic disorders seen during pregnancy [128], with an incidence rate of up to 28% [119]. In 2017, 204 million people worldwide were diagnosed with GDM, with projections to reach 308 million by 2045 [119]. In the USA, the prevalence increased from 4.6% in 2006 to 8.2% ten years later [129]. Mainland China’s incidence of GDM was 14.8% between 2010 and 2017 [130]; in the Gulf Cooperation Council (GCC) countries, the prevalence increased by 4% during the last 20 years to reach 15.9% today. In those countries, the women most at risk were those who were overweight or obese, over 30 years old and those delivering their baby through C-section [131]. Pregnant women in Europe experience have a significant incidence of GDM (11%), with pregnant women in Eastern European countries experiencing the highest prevalence (31.5%) [132].

### 8.2. Association between Particulate Matter (PM) and Gestational Diabetes (GDM)

GDM’s well-defined risk factors cannot fully explain its rapid increase worldwide in recent decades. This has led to a growing interest in experimental and epidemiology studies to gain insight into the correlation between pollutant exposure and GDM [133]. Unfortunately, the results are inconclusive, and no direct correlation has been established. Ambient air pollution exposure has been hypothesized to increase insulin resistance inciting metabolic dysfunction, and different pathways have been proposed to explain the association.

Firstly, exposure to PM_2.5_ and NO could accelerate β-cell dysfunction in already susceptible pregnant women [127]. Secondly, PM could lead to altered leptin and adiponectin concentrations, regulators of neurohormonal metabolic control [127]. The reduction of adiponectin and leptin has been demonstrated in a study involving long-term exposure to PM_2.5_ in C57BL/6 mice [61]. Lavigne et al. demonstrated that exposure during pregnancy to PM_2.5_ resulted in an 11% increase in adiponectin levels, and NO_2_ exposure resulted in a 13% increase in adiponectin levels [134]. The proposed mechanism causing the imbalance of these adipokines involves an inflammatory response in adipose tissues following PM exposure. Maternal exposure to air pollution has also been linked to increased local or systemic inflammatory responses, causing complications during pregnancy [127].

Furthermore, metals, sulfur and organic components comprising PM have also been associated with stimulating oxidative damage through ROS production in the mitochondria, formation of AGEs, activation of protein kinase C, reduction of GLUT4 and translocation of nuclear factor kappa B (NF-κB) subunit 1 into the nucleus, thereby generating an inflammatory response that impacts insulin signaling [127]. Air pollution could also increase gut permeability by altering the gut microbiota, thereby inducing the transport of inflammatory mediators from the gut into the bloodstream [127]. Additionally, a positive association has been found between PM_2.5_ exposure up to 12 weeks prior to conception and fasting glucose levels, thus increasing the risk for GDM in 11,639 women in China from 2016 to 2017 [124].

Furthermore, it has been postulated that pesticides increase diabetes by increasing the accumulation of acetylcholine which causes an increase in glucose [135].

A positive association was found between the total serum concentrations of nine PCB congeners and, more significantly, with eight PBDE congeners and GDM in a study involving seventy pregnant Iranian women with no family history of diabetes between the ages of 16 and 40 years in their third trimester [136]. Another study assessed the effect of exposure to PCBs and pesticides (hexachlorobenzene, HCB, and a DDT metabolite (dichlorodiphenyldichloroethane, DDE)) in the first trimester in 939 women in Greece [137]. The study found a strong positive association between PCB serum concentration and the probability of GDM. No statistically significant results were found for DDE and HCB. A prospective cohort study involving 2292 pregnant women in their 8th to 11th week of gestation without significant comorbidities demonstrated an association between serum levels of heavily chlorinated PCBs and PBDEs (47 and 154) and GDM [29].

Conversely, a study measured serum concentrations of certain POPs in the first trimester of 1274 Canadian pregnancies with no pre-existing diabetes and found no evidence of an association between PCBs and GDM [135]. An inverse relationship between urine concentrations of pesticides (dimethylphosphate (DMP) and dimethylthiophosphate (DMTP)) and GDM was observed, a result as perplexing as it is unique, which has not been reproduced in other studies to date.

The wide disparity of results is the most significant limitation of these studies on GDM. Gestational diabetes has multifactorial risk factors, and it is hard to isolate all confounding factors to determine the direct effects of exposure of different concentrations of the POPs. Adding further complication to identifying environmental pollutants as causative, a mixture of the many comorbidities, family history of T2D, genetic predisposition, lifestyle, physical activity status, overweight/obesity status and smoking habits all affect the incidence of GDM.

## 9. Metabolic Syndrome

Metabolic syndrome (MetS) refers to a cluster of disorders that increase adverse cardiometabolic outcomes. MetS is diagnosed when at least three of the following criteria are met: a BMI over 30 kg/m^2^, increased blood triglycerides, low high-density lipoprotein (HDL) levels, increased blood pressure and elevated blood glucose levels [26,138]. This condition is increasingly concerning as it represents a risk factor for chronic conditions such as type 2 diabetes and cardiovascular disease.

Metabolic syndrome has seen a surge in prevalence in recent decades. Data from 51,371 participants were used to estimate the prevalence of metabolic syndrome from 1988 to 2012 in the USA. The prevalence increased from 25.3% in 1988 to 34.2% in 2012. Indeed, it is estimated that more than one third of US adults satisfy the MetS requirements, with the greatest burden borne by non-Hispanic black adults and those of low socioeconomic status. Interestingly, the increase in prevalence cannot be attributed solely to the rising cases of obesity, as the prevalence of MetS among non-obese individuals remained stable across the time period studied [139]. In the Asia-Pacific region, specifically China, South Korea and Taiwan, it was seen that between the years of 1993 and 2009 there was a distinct increase in metabolic syndrome prevalence [140]. Furthermore, the prevalence of MetS increased rapidly with age. With the aging population, additional rises in MetS are predicted and, thus, an increase in associated chronic diseases [139].

With growing urbanization and drastically increasing air pollution, it is difficult to single out exposure to air pollutants from other human interactions with the environment. As epigenetic evidence suggests, there is a substantial influence of the environment on the human genome, with long-term exposure eventually causing epigenetic changes such as DNA methylation. There is growing research suggesting that epigenetic variation influenced by air pollution results in genetic susceptibility to metabolic dysregulation and later development of type 2 diabetes mellitus. Understanding this linkage can assist in identifying biomarkers associated with metabolic syndrome and could provide indications for early intervention and treatment [26].

In a study conducted in the Czech Republic, 200 blood samples from children living in two separate geographic regions were compared. The study found that samples from Oztrava Province (a markedly polluted region) were found to have a 36% difference in methylation of CpG sites versus those children from the Prachatice region (a pollution-controlled region). This study found a negative association between DNA methylation and exposure to air pollutants (B[a]P, benzene, NO2, PM2.5, PM10 and metals: arsenic, cadmium, nickel and lead) [141]. These findings are reinforced by a randomized, double-blind crossover study conducted in China of 36 participants who were provided with either real or fake air purifiers, where those provided fake purifiers demonstrated DNA methylation of CpG sites, involving glucose and lipid metabolism, and insulin resistance, thus suggesting the role of air pollution in the development of cardiac and metabolic pathologies [142].

Methylation of DNA has been shown to contribute to cardiovascular complications, blood glucose elevations and increased adipose tissue, all of which underlie metabolic syndrome development.

## 10. Conclusions

The impact of pollution on metabolic disorders has garnered huge interest due to the increasing incidence of these disorders that cannot solely be explained by currently established risk factors. Several mechanisms have been highlighted throughout this paper with regard to the linkage between pollutants and obesity, T1D, T2D, GDM and MetS. However, research has shown inconsistent results with regard to direct pathogenesis. This could be due to several causes. Firstly, these diseases are multifactorial, so it is difficult to isolate a single risk factor. Secondly, many studies attempted to determine the impact of isolated atmospheric compounds but, by not taking into consideration their compounding effects, a clear understanding of their overall involvement is lacking. Finally, by attempting to reduce the effects of comorbidities, smaller sample sizes were achieved which reduced the robustness and reproducibility of the findings. Future research is required to further our understanding of the effects of pollutants and to implement the necessary changes to reduce pollution, improve health outcomes and protect future generations.

## Figures and Tables

**Table 1:** Sources and impacts on the endocrine system of common organic pollutants (POPs).

Pollutant	Source of Pollutant	Impact on Endocrine System
Polychlorinated biphenyls (PCBs)	Industrial chemicals (mainly electrical products) [19,34]	Disrupts thyroid homeostasis Estrogen and androgen agonistic and antagonistic properties [19,35]
Dichlorodiphenyltrichloroethane (DDT)	Pesticide Currently used to rid vectors of disease in parts of the world [30]	Antiandrogenic properties Hypothesized xenoestrogen properties Hypothesized to work in additive way with other EDC pollutants [36,37]
Polycyclic aromatic hydrocarbons (PAHs)	Synthetic chemical used in industries, food preparation and cigarettes [19,38]	Agonist and antagonist of estrogen [19,38]
Bisphenol A (BPA)	Industrial chemical used for plastic and resin [19,38]	Xenoestrogen: binds and activates estrogen receptor Antiandrogen: binds and blocks androgen receptor Modifies steroid synthesis and affects its circulating concentration Affects peroxisome proliferator-activated receptors (PPARs), altering regulation of gene expression Disturbs thyroid and glucocorticoid signaling [19,39]
Phthalates	Industrial chemical used in plastics [19,38]	Strong antiandrogenic properties (weak estrogenic properties) [40]
Polybrominated diphenyl ethers (PBDEs)	Class of fire-retardant chemicals [41]	Disruption of thyroid hormone regulation by binding to thyroid nuclear and transporter receptors Agonist and antagonist of estrogen, androgen and progesterone receptors [19,41,42]

**Table 2:** Stockholm Convention annexes [28].

Annex A: Elimination	Annex B: Restriction	Annex C: Unintentional Production
Aldrin Chlordane Chlordecone Decabromodiphenyl ether Dicofol Dieldrin Endrin Heptachlor Hexabromobiphenyl Hexabromocyclododecane Hexabromodiphenyl ether and heptabromodiphenyl ether Hexachlorobenzene Hexachlorobutadiene Alpha hexachlorocyclohexane Beta hexachlorocyclohexane Lindane Mirex Pentachlorobenzene Pentachlorophenol and its salts and esters Polychlorinated biphenyls Polychlorinated naphthalenes Perfluorooctanoic acid, its salts and PFOA-related compounds Short-chain chlorinated paraffins Technical endosulfan and its related isomers Tetrabromodiphenyl ether and pentabromodiphenyl ether Toxaphene	DDT Perfluorooctane sulfonic acid, its salts and perfluorooctane sulfonyl fluoride	Hexachlorobenzene Hexachlorobutadiene Pentachlorobenzene Polychlorinated biphenyls Polychlorinated dibenzo-p-dioxins Polychlorinated dibenzofurans Polychlorinated naphthalenes

**Table 3:** Classifications of pesticides.

Types of Compounds	Examples
Organochlorine compounds	DDT Chlordane Methoxychlor Lindane
Organophosphate compounds	Chlorpyrifos Carbamates Pyrethroids

## Data Availability

No novel data were produced in the writing of this review article.
